# Association of cartilage $T_{1\rho \;}$ and $T_{2}$ relaxation time measurement with hip osteoarthritis progression: A 5-year longitudinal study using voxel-based relaxometry and Z-score normalization

**DOI:** 10.1016/j.ocarto.2024.100538

**Published:** 2024-10-28

**Authors:** Rafeek Thahakoya, Koren E. Roach, Misung Han, Rupsa Bhattacharjee, Fei Jiang, Johanna Luitjens, Emma Bahroos, Valentina Pedoia, Richard B. Souza, Sharmila Majumdar

**Affiliations:** a Department of Radiology and Biomedical Imaging, University of California San Francisco, CA, USA; b Department of Biomedical Engineering, Schulich School of Engineering, University of Calgary, Calgary, Alberta, Canada; c Department of Radiology, Cumming School of Medicine, University of Calgary, Calgary, Alberta, Canada; d McCaig Institute for Bone and Joint Health, Cumming School of Medicine, Calgary, Alberta, Canada; e Department of Epidemiology and Biostatistics, University of California San Francisco, San Francisco, CA, USA; f Department of Physical Therapy and Rehabilitation Science, University of California San Francisco, San Francisco, CA, USA

**Keywords:** Articular cartilage, Hip osteoarthritis, VBR analysis, Longitudinal study

## Abstract

**Objective:**

To study the longitudinal changes of cartilage $T_{1\rho \;}$ and $T_{2}$ relaxation time measurements in hip-OA patients.

**Methods:**

A calibration study compared two scanner data, Scanner-1 (GE Discovery MR750 3.0T) with unilateral acquisition protocol and Scanner-2 (GE Signa Premier 3.0T) with bilateral acquisition protocol, using nine subjects(average age ​= ​40.33 ​± ​13.53 years, 5 females), including one hip-OA subject. Quantified parameters from the Scanner-2 were adjusted using voxel-based relaxometry(VBR) and Z-score normalization to reduce the inter-scanner variability. Eighteen hip-OA Subjects (age ​= ​53.11 ​± ​14.96 years, 12 females) were recruited to the longitudinal variability study from 2016, comprising five assessments at 1-year intervals. Baseline to 3rd-year data used unilateral acquisition with Scanner-1, while 4th-year data used bilateral acquisition with Scanner-2. A linear mixed-effects model(LME) assessed trajectory analyses, with acquisition year, age, sex, body mass index(BMI), and Kellgren-Lawrence(KL) score as predictor variables and cartilage mean $T_{1\rho \;}$ and $T_{2}$ values as outcomes.

**Results:**

VBR analysis after Z-score normalization showed that only a few of the whole cartilage voxels had significant differences in $T_{1\rho \;}($femur-2.36 ​% and acetabular-3.23 ​%) and $T_{2}$ (femur-2.30 ​% and acetabular-2.94 ​%) values between the scanners. The LME analysis showed that the BMI predictor variable was significantly correlated with the femur $T_{1\rho \;}$ (p ​< ​0.0001) and $T_{2}$ (p ​< ​0.0001) and acetabular $T_{1\rho \;}$ (p ​< ​0.0001) and $T_{2}$ (p ​< ​0.0001) cartilage region.

**Conclusion:**

The calibration study demonstrated the effectiveness of VBR and Z-score normalization in reducing inter-scanner variability. The longitudinal study revealed a significant correlation between $T_{1\rho \;}$ and $T_{2}$ values of the cartilage and BMI; also the $T_{1\rho \;}$ and $T_{2}$ values increased over time in some of the cartilage subregions.

## 1 Introduction

Hip osteoarthritis(OA) is the second most affected joint disease and causes pain and disability among the elderly [1]. The articular cartilage degeneration in the weight-bearing joints is the onset of OA [2]. Traditionally, conventional radiographs have been employed in the diagnosis and treatment planning of hip-OA based on the assessment of joint space narrowing and the presence of osteophytes [3,4]. The severity of the disease is often characterized using the well-established Kellgren-Lawrence(KL-score) clinical score [5]. However, this radiograph-based analysis lacks soft tissue information, leading to a low sensitivity to the changes that result from OA progression [2]. Incorporating imaging biomarkers to identify the early and subtle changes in soft tissue is essential for improving OA diagnosis and treatment planning.

Conventional magnetic resonance imaging(MRI) techniques are widely used to analyze morphological changes in articular cartilage due to its soft tissue contrast [6,7]. However, early biochemical changes of articular cartilage before structural damage can be evaluated with the help of compositional imaging techniques such as $T_{1\rho \;}$ and $T_{2}$ relaxation time measurements of cartilage [8,9]. Multiple cross-sectional and longitudinal studies reported increased $T_{1\rho \;}$ and $T_{2}$ values in hip cartilage are associated with disease progression [[10], [11], [12], [13]]. Recently developed automatic voxel-based relaxometry(VBR) [14,15] employs a method centered around aligning all subjects onto a unified reference space and is validated as a tool to provide robust and reliable quantification of hip cartilage. It helps to assess local patterns of quantification maps in the cartilage region and the differences between different subject groups [11].

A few longitudinal studies have shown the association of baseline $T_{1\rho \;}$ and $T_{2}$ relaxation times in hip cartilage lesion progression [11,14]. One recent study reported a significant increase of baseline hip femoral cartilage $T_{1\rho \;}$ and $T_{2}$ values in subjects who experienced femoral cartilage lesion progression using traditional ROI analysis and VBR approach [11]. VBR was reported to be more sensitive to local patterns of $T_{1\rho \;}$ and $T_{2}$ increase in the cartilage region. In this study, the results indicated age, body mass index(BMI), presence of baseline hip-OA and female sex showed a positive trend for OA progression. However, this study was limited to baseline and 18-month follow-up periods. Hence, conducting a longitudinal study with an extended follow-up period is essential to establish a more robust correlation between $T_{1\rho \;}\text{and}\;T_{2}$ values and the hip-OA progression.

Major hardware or software changes with MRI scanners can occur during longitudinal studies and variability might affect the resultant analysis [16]. An appropriate correction method is required to achieve unbiased $T_{1\rho \;}$ and $T_{2}$ measurements. Recent studies showed the Z-score-based normalization technique is more effective for correcting the scaling factor to the $T_{1\rho \;}$ and $T_{2}$ measurements due to the scanner or coil updates [17,18]. A recent study showed a significant improvement in intra-subject comparability of $T_{1\;}$ (ICC of 0.11 vs. 0.78) and $T_{2\;}$ (ICC of 0.35 vs. 0.83) when using Z-scores across three scanners [20]. Therefore, such normalization methods can be useful for eliminating the bias due to the scanner coil upgrade or multicenter acquisition.

The aims of the current study were to 1) Reduce the inter-scanner variability of $T_{1\rho \;}$ and $T_{2}$ quantification between MR scanners and 2) Evaluate the changes in femoral and acetabular cartilage $T_{1\rho \;}$ and $T_{2}$ relaxation time measurements for patients with early to moderate hip-OA over 5-years.

## 2 Materials and methods

Two separate studies were included: (i) Calibration study (ii) Longitudinal variability study.

### 2.1 Calibration study

A calibration study was performed to reduce the inter-scanner variability between the two scanners involved in the longitudinal variability study.

**Study Population and Data Selection:** Nine subjects were involved in the calibration study(age ​= ​40.33 ​± ​13.53 years, 5 females), including one OA subject with KL-score 1 for both hips. The inclusion criteria of the subject selection include(1) Subjects over 18 years(2) Subjects with no history of hip or knee surgery and no contraindications for MRI.

**MRI Acquisition:** The images were acquired using unilateral acquisition protocol on a GE Discovery MR750 3.0T scanner (GE Healthcare, Waukesha, WI) named as Scanner-1 with a 32-channel phased-array cardiac coil(Invivo Corp., Amsterdam, Netherlands) positioned over the left and right hip sequentially. The scanning started with the left side, followed by the right hip and the participant arrived 30 ​min before the scan. The same subjects were imaged using simultaneous bilateral hip imaging protocol on a GE Signa Premier 3.0T MR Scanner (GE Healthcare, Waukesha, WI) named as Scanner-2 with a 30-channel adaptive image receive(AIR) anterior array coil and a 60-channel spine posterior-array coil embedded into the table(GE Healthcare, Waukesha, WI). The subjects were imaged using Scanner-1 and Scanner-2 in 2022 with a maximum gap of one month between the acquisition. The subjects were positioned in a supine posture and feet-first inside the scanner. Magnetization-prepared angle-modulated partitioned k-space spoiled gradient echo snapshots(MAPSS) sequence was acquired for cartilage $T_{1\rho \;}$ and $T_{2}$ assessments [19]. The detailed scanning protocol for both unilateral and bilateral acquisition is summarized in Table 1.

**Table 1:** MRI acquisition parameters and scanner details.

Parameters	Scanner-1 (Unilateral)	Scanner-2 (Bilateral)
Scanner used	GE Discovery MR750 3.0T scanner (GE Healthcare, Waukesha, WI)	GE Signa Premier 3.0T MR scanner (GE Healthcare, Waukesha, WI)
Coils used	32-Channel phased-array cardiac coil (Invivo Corp., Amsterdam, Netherlands)	30-Channel adaptive image receive (AIR) anterior array coil and a 60-channel spine posterior-array coil (GE Healthcare, Waukesha, WI)
Sequence name	Hip MAPSS sagittal	Hip MAPSS sagittal
Acquisition time	11 ​min	16 ​min 30 ​s
Acquisition Matrix	256 × 128	256 × 128
TR (per view)	5.2	5.2
TSLs (ms)	0, 15, 30, 45	0, 15, 30, 45
TEs (ms)	0, 10.4, 20.8, 41.6	0, 10.4, 20.8, 41.6
FOV (cm ​× ​cm)	14 × 14	14 × 14
Slice thickness (mm)	4	4
ARC Acceleration factor	2 × 1 (k_y_ x k_z_)	2 × 2 (k_y_ x k_z_)
Y-FOV Oversampling factor	100 ​%	40 ​%
Number of slices	20	60

**Image Processing**: All post-processing techniques were performed using an in-house program developed in MATLAB integrated with the elastix toolbox for the non-rigid image registration(version R2019a, The MathWorks Inc., Natick, MA, USA).

***Cartilage Segmentation and***$T_{1\rho \;}and\;T_{2}$***Quantification***: From the acquired bilateral MAPSS images(sagittal view) from Scanner-2, the left and right hip images were automatically divided into left and right image stacks. In the case of Scanner-1 unilateral acquisition images, the left and right-side hip images were acquired separately. The following post-processing steps were common for all the images acquired using both scanners [20]. The acetabular and femoral hip cartilage were automatically segmented using a previously validated atlas-based approach [14]. Briefly, the first echo MAPSS images were registered to a previously defined reference atlas using a non-rigid elastix registration method. The registration transformation was applied to the remaining echo images. The reference cartilage region of interest(ROI) of four slices near the hip center was generated using a semi-automated segmentation algorithm based on Bezier splines and edge detection.

The $T_{1\rho \;}$ mapping was obtained by fitting the images from multiple TSLs using Levenberg-Marquardt mono-exponential equation without considering the noise level [21]:

(1) $$S\left(\text{TSL}\right)=S_{0}\;e^{-\left(TSL/T_{1\rho }\right)}$$

Similarly, the $T_{2}$ mapping was obtained by fitting the multiple TEs corresponding to the images by Levenberg-Marquardt mono-exponential equation without considering the noise level [21]:

(2) $$S\left(T2\right)=S_{0}\;e^{-\left(TE/T_{2}\right)}$$

While bi-exponential fitting can distinguish between tightly and loosely bound water in cartilage, monoexponential fitting still offers valuable insights. With only four data points for fitting T₁ρ and T₂, bi-exponential fitting is challenging. The fitted $T_{1\rho \;}$ and $T_{2}$ maps were used for the quantitative evaluation after the segmentation. The reference atlas-based femur, acetabular cartilage masks, and subregion segmentations were superimposed on $T_{1\rho \;}$ and $T_{2}$ maps of each patient data. The cartilage subregions(Fig. 2(B)) are: R_2_ as posterior, R_3_ as posterior-superior, R_4_ as superior, R_5_ as anterior-superior, R_6_ as anterior, and R_7_ as anterior-inferior cartilage regions [20]. In the eight subregions($R_{1}$-$R_{8}$), 5 subregions were selected from the femur cartilage($R_{2}$-$R_{6}$) and 4 subregions($R_{2}$-$R_{5}$) from acetabular cartilage and excluded subregions with less than 50 pixels overall segmented slices($R_{1}{,R}_{7}$ and $R_{8}$ for the femur and $R_{1}$, ${\;R}_{6}{,R}_{7\;}$ and $R_{8}$ for the acetabulum cartilage) [11,22]. The upper threshold of 100 ​ms and 80 ​ms were used for $T_{1\rho \;}$ and $T_{2}$ maps respectively to remove the effects by fluid or partial volume [20].

***ROI analysis:*** In the calibration study, T₁ρ and T₂ values in the hip cartilage of volunteers were compared between Scanner-1 and Scanner-2 using the mean ROI selection method, which averaged values across all pixels in the femoral and acetabular cartilage regions.

***Voxel-based Relaxometry analysis:*** VBR technique was used to compare local patterns of $T_{1\rho \;}$ and $T_{2}$ relaxation time measurements between Scanner-1 and Scanner-2 on a voxel-basis [14].

***Z-score Normalization:*** The mean and standard deviation of $T_{1\rho \;}$ and $T_{2}$ values obtained from VBR analysis were used for scanner specific(Scanner-1 and Scanner-2) Z-score generation using the formulas:

(3) $$Z_{i_{\text{scanner}1}}=\frac{T_{i\;\text{scanner}1}-{\text{MeanT}}_{\text{scanner}1}}{\sigma_{\text{scanner}1}}$$

(4) $$Z_{i_{\text{scanner}2}}=\frac{T_{\text{iscanner}2-{\text{MeanT}}_{\text{scanner}2}}}{\sigma_{\text{scanner}2}}$$

Where $Z_{i}$ is the Z-score of $i^{\text{th}}$ voxel, $T_{i\;}$ is the $T_{1\rho \;}\text{or}\;T_{2}$ value of $i^{\text{th}}$ voxel, MeanT represents the mean $T_{1\rho \;}\text{or}\;T_{2}$ values of the Scanner-1 or Scanner-2 groups and σ represents the standard deviation of the $T_{1\rho \;}\text{or}\;T_{2}$ values of the Scanner-1 or Scanner-2 groups.

All Z-score values beyond ±4rangewerethresholded to +4 or −4, respectively [22]. The Z-score maps were generated and qualitatively analyzed the map patterns of cartilage $T_{1\rho \;}\text{and}\;T_{2}$ values and quantitatively analyzed with the help of VBR. The calibration factor derived from(3) and(4) is:

(5) $$T_{i\;\text{calib}\_\text{factor}}=\left(\left(\frac{T_{i\;\text{scanner}1}-{\text{MeanT}}_{\text{scanner}1}}{\sigma_{\text{scanner}1}}\right)*\sigma_{\text{scanner}2}\right)$$

The observed calibration factor $T_{i\;\text{calib}\_\text{factor}}$ from equation (5) was used to update the $T_{1\rho \;}$ and $T_{2}$ values acquired bilaterally from Scanner-2 using the expression:

(6) $$T_{i\;\text{calib\_scanner}2}=T_{i\;\text{calib}\_\text{factor}}+{\text{MeanT}}_{\text{scanner}2}$$

### 2.2 Longitudinal variability study

In the longitudinal variability study, subjects were imaged using unilateral acquisition protocol on Scanner-1 from baseline to 3rd-year follow-up. During the 4th-year follow-up, same subjects were images using bilateral acquisition protocol on Scanner-2.

**Study Population and Data Selection**: Eighteen hip-OA subjects(age ​= ​53.11 ​± ​14.96 years, 12 females) were recruited from a previous study [22]. The inclusion criteria of the subject selection included:(1) Hip-OA subjects having KL-score less than or equal to 3(KL-score>3 considered advanced-OA). The KL-score was assessed from radiographs acquired at the baseline by an experienced musculoskeletal radiologist with 3-years of training. (2) Subjects age above 18-years. (3) Absence of intra-articular injection in the past 6 months of recruitment. (4) Subjects not having previous history of hip or knee surgery and no contraindications for using MRI.

**Fig. 1:**
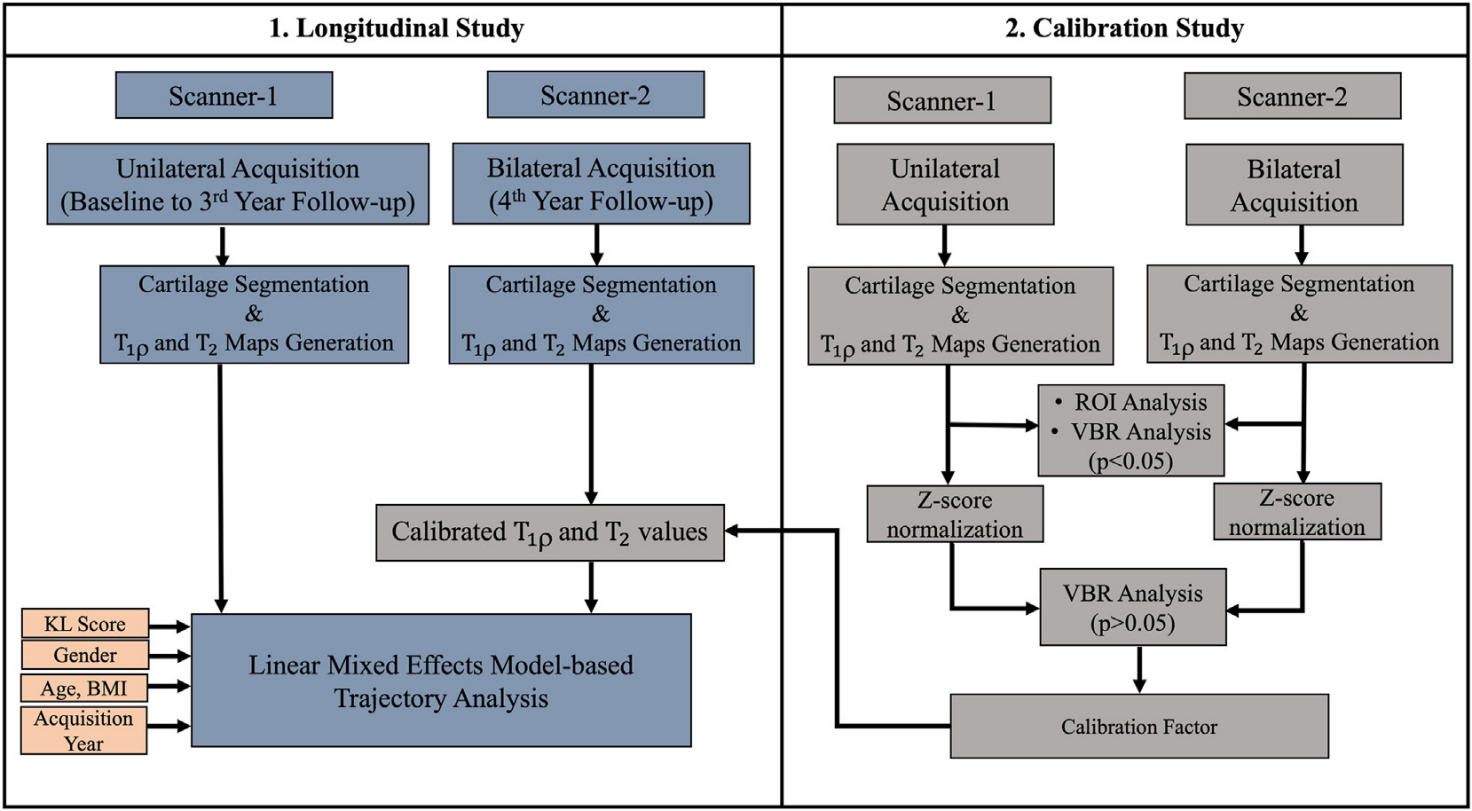
Flow diagram of the processing pipeline. Scanner-1 is a GE Discovery MR750 3.0T scanner and Scanner-2 is a GE Signa Premier 3.0T MR Scanner.

**MRI Acquisition:** In the longitudinal study, MR images were acquired for all patients using unilateral acquisition protocol on Scanner-1 with a 32-channel phased-array cardiac coil(Invivo Corp., Amsterdam, Netherlands) positioned sequentially over the left and right hip. The images were acquired at baseline, 1st-year follow-up, 2nd-year follow-up and 3rd-year follow-up from a previous study and were imaged yearly from 2016 [22]. During the 4th-year follow-up, the same subjects were imaged using simultaneous bilateral hip imaging protocol on Scanner-2 with a 30-channel adaptive image receive(AIR) anterior array coil and a 60-channel spine posterior-array coil embedded into the table(GE Healthcare, Waukesha, WI). All the baseline to 3rd-year follow-up study, subjects were segmented and the mean $T_{1\rho \;}$ and $T_{2}$ values were obtained using the post-processing steps explained in the section ‘*Cartilage Segmentation and* $T_{1\rho \;}and$ $T_{2}$ *Quantification’* [14]. In the case of the 4th-year follow-up study, the obtained $T_{1\rho \;}$ and $T_{2}$ values were modified using the $T_{i\;\text{calib}\_\mathrm{factor}}$ scaling factor derived from equation (5). The overall processing pipeline of the study is illustrated in Fig. 1.

**Fig. 2:**
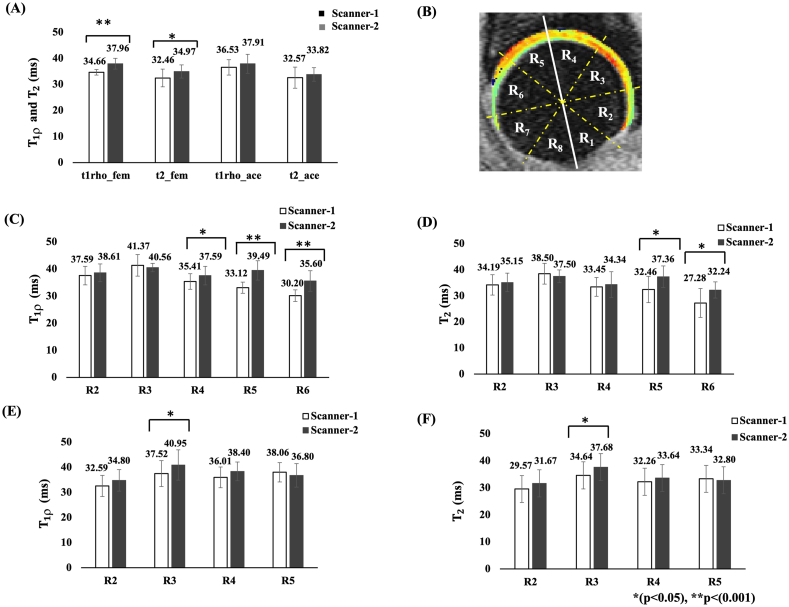
Image(A) shows the bar graph representation of mean $T_{1\rho \;}$ and $T_{2}$ measurements of the femur and acetabular cartilage using ROI analysis(calibration study). Image(B) represents the divisions of the femur and acetabular cartilage subregions. The solid white line represents a reference line parallel with the femoral neck; the spherical shape is divided into eight equal subregions with 45° each represented by the yellow dashed line, and the regions are labeled as R_1_–R_8_. From this, R_2_–R_7_ are considered in the current study. R_2_:posterior, R_3_:posterior-superior, R_4_:superior, R_5_:anterior-superior, R_6_:anterior and R_7_:anterior-inferior cartilage regions. Image(C) and (D) shows the representation of mean $T_{1\rho \;}$ and $T_{2}$ values of femur cartilage sub regions($R_{2}$-$R_{6}$) whereas image(E) and (F) represent the mean $T_{1\rho \;}$ and $T_{2}$ values of acetabular cartilage subregions($R_{2}$-$R_{5}$). t1rho_fem: mean $T_{1\rho \;}$ values of femoral cartilage, t2_fem: mean $T_{2}$ values of femoral cartilage, t1rho_ace: mean $T_{1\rho \;}$ values of acetabular cartilage, t2_ace: mean $T_{2}$ values of acetabular cartilage.

The mean $T_{1\rho \;}$ and $T_{2}$ measurements of the hip femur and acetabular cartilage were evaluated at baseline, 1st-year, 2nd-year and 3rd-year follow-up. The 4th-year follow-up quantification was adjusted from the original $T_{1\rho \;}$ and $T_{2}$ values using the scaling factor derived from the calibration study. For evaluating the changes in cartilage $T_{1\rho \;}$ and $T_{2}$ measurements after calibration was obtained by subtracting the mean values at follow-up from the initial baseline values. The percentage change was calculated based on 3rd-year and 4th-year follow-up time as follows:

(7) $$\text{Percentage}\;\text{change}=\left[\frac{\text{Follow}-\text{up}\;T_{1\rho \;}\text{or}\;T_{2}\;\text{values}\;-\;\text{Initial}\;T_{1\rho \;}\text{or}\;T_{2}\;\text{values}}{\text{Initial}\;T_{1\rho \;}\text{or}\;T_{2}\;\text{values}}\right]\times 100$$

### 2.3 Statistical analysis

Paired Student's t-test was used in ROI and VBR analysis for evaluating the difference in cartilage mean $T_{1\rho \;}$ and $T_{2}$ values between Scanner-1 and Scanner-2. A p-value of 0.05 was selected as the significant threshold level. VBR p-maps were generated and overlayed on the hip cartilage region to display the voxels with significant differences between the scanners.

The different time point trajectory analyses of hip-OA patients were assessed using a linear mixed effect (LME) model adopted from the R Development Core Team, v4.2.3 with the “lmertest”, and “lme4” packages. Initially, a null model was generated and compared with a full model where the acquisition year, sex, age, BMI and KL-score were selected as predictor variables in the mixed effect model. In these two models, hip femur and acetabular mean $T_{1\rho \;}$ and $T_{2}$, sub-region mean $T_{1\rho \;}$ and $T_{2}$ values were considered outcome variables.

The fixed-effects formula was:

(8) $$\text{Mean}\;T_{1\rho \;}\;\text{and}\;T_{2}\;\text{values}\;\text{of}\;\text{the}\;\text{femur}\;\text{and}\;\text{acetabular}\;\text{cartilage}\;\text{or}\;\text{sub}-\text{region}\;∼\;\text{acquisition}\;\text{year}+\text{sex}+\text{age}+\text{KL}-\text{score}+\text{BMI}-1$$

and random-effect formula was:

(9) random ​= ​∼1 | Participant-ID

## 3 Results

### 3.1 Calibration study

In the calibration study, one subject was excluded due to its poor image registration. The ROI analysis results show a significant difference in the femur mean $T_{1\rho \;}$ (p ​< ​0.001) and $T_{2}($p ​= ​0.024) values between Scanner-1 and Scanner-2. The subregion mean $T_{1\rho \;}$ values of femoral cartilage region show a significant difference in $R_{4}($p ​= ​0.002), $R_{5}($p ​< ​0.0001) and $R_{6}($p ​= ​0.002) between scanners, whereas the mean $T_{1\rho \;}$ value of acetabular cartilage region showed the significant difference in one subregion $R_{3}$ (p ​= ​0.02). The subregion mean $T_{2}$ values of the femoral cartilage region showed a significant difference in $R_{5}($p ​= ​0.002) and $R_{6}($p ​= ​0.008), whereas the mean $T_{2}$ value of acetabular cartilage region showed a significant difference in one subregion $R_{3}($p ​= ​0.0372).

**Fig. 3:**
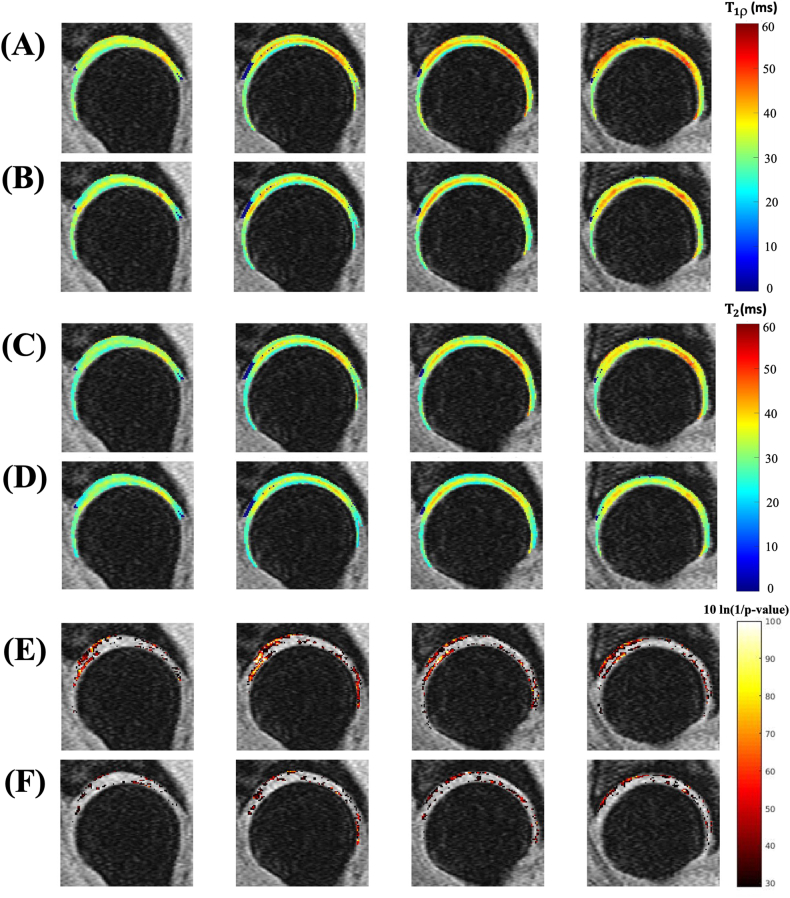
Visualization of hip cartilage images with VBR results. Eight subjects(including 16 hips from both the left and right sides) hip images were included in the VBR analysis, utilizing four central slices from the sagittal view. Image(A) is the mean $T_{1\rho \;}$ value observed from Scanner-1 and image(B) showed the mean $T_{1\rho \;}$ value from Scanner-2. Image(C) and (D) showed the mean $T_{2}$ value observed from Scanner-1 and Scanner-2 respectively. Images(E) and (F) represent the p-value maps(logarithmic scale) corresponding to mean $T_{1\rho \;}$ and $T_{2}$ values of both the scanners.

The VBR analysis based on eight subjects showed that, before Z-score normalization, 29.68 ​% of the femur cartilage and 23.9 ​% of the acetabular cartilage had significant differences in mean $T_{1\rho \;}$ values between the scanners (Fig. 3(E)). Also, the VBR analysis showed that 17.65 ​% of the femur cartilage and 10.61 ​% of the acetabular cartilage voxels significantly differ in $T_{2}$ values between the scanners (Fig. 3(F)). The $T_{2}$ measurements showed a comparatively lesser number of voxels have statistically significant difference than $T_{1\rho \;}$ values. The trend was like the corresponding ROI analysis results shown in Fig. 2. Hence, the ROI and VBR analysis results showed a statistically significant difference between Scanner-1 and Scanner-2.

**Supplementary figure 1:**
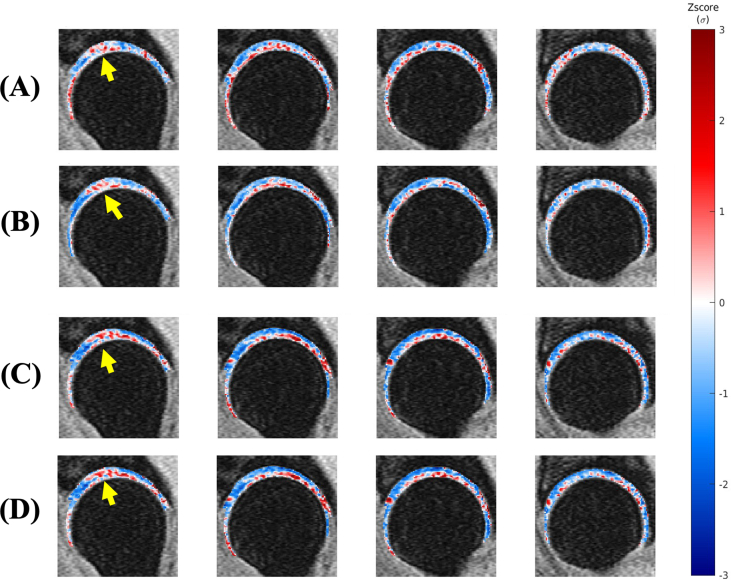
The Z-score maps based on the mean and standard deviation of $T_{1\rho \;}$ (A) and $T_{2}($ C) values of Scanner-1 and Z-score maps based on the mean and standard deviation of $T_{1\rho \;}$ (B) and $T_{2}($ D) values of Scanner-2. The yellow arrow shows the presence of the focal lesion was qualitatively observed in the Z-score maps of both scanners.

The generated Z-score map patterns were qualitatively analyzed after thresholding, based on the $T_{1\rho \;}\text{and}\;T_{2}$ values. Example of one Z-score map from a healthy volunteer (age ​= ​44years, side ​= ​right hip) were shown in Supplementary Fig. 1 based on the mean and standard deviation of $T_{1\rho \;}$ values of Scanner-1 (Supplementary Fig. 1(A)) and Scanner-2 (Supplementary Fig. 1(B)) and the mean and standard deviation of $T_{2}$ values of Scanner-1 (Supplementary Fig. 1(C)) and Scanner-2 (Supplementary Fig. 1(D)). From these maps, the focal lesion presence(yellow arrow) was observed in Scanner-1 and Scanner-2 corresponding to $T_{1\rho \;}\text{and}\;T_{2}$ values indicating the reduced inter-scanner variability bias between the scanners.

**Fig. 4:**
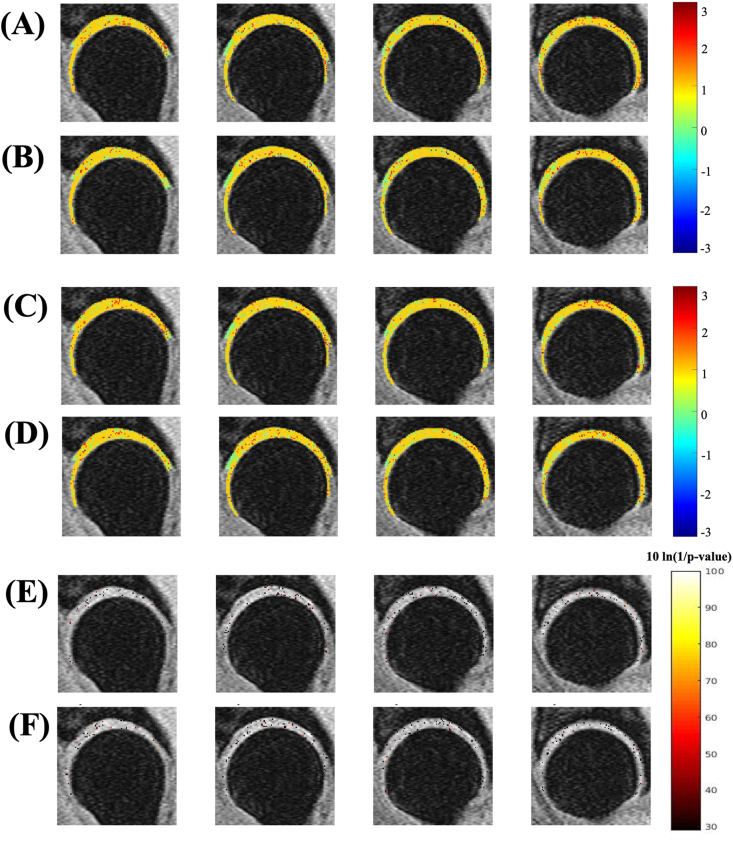
VBR results after calibration correction. Image(A) shows the Z-score maps of Scanner-1 and image(B) shows the Z-score maps of Scanner-2, which correspond to $T_{1\rho \;}$ values. Images(C) and(D) show the Z-score maps of Scanner-1 and Scanner-2 correspond to $T_{2\;}$ values. Image(E) represents the p-value maps(logarithmic scale) corresponding to $T_{1\rho \;}$ values and image(F) represents the p-value maps corresponding to $T_{2\;}$ values.

The VBR results after Z-score normalization are shown in Fig. 4. The VBR p-maps after Z-score normalization show that only 2.36 ​% and 3.23 ​% of the whole cartilage voxels have a significant difference in $T_{1\rho \;}$ between the scanners for the femur and acetabular regions, respectively. Also, the VBR p-maps analysis after Z-score normalization shows that 2.30 ​% and 2.94 ​% of the whole cartilage voxels displayed significant differences in $T_{2}$ between the scanners for the femur and acetabular regions, respectively. That means the VBR analysis after Z-score normalization showed a very small number of voxels displayed a statistically significant difference between Scanner-1 and Scanner-2.

### 3.2 Longitudinal variability study

**Fig. 5:**
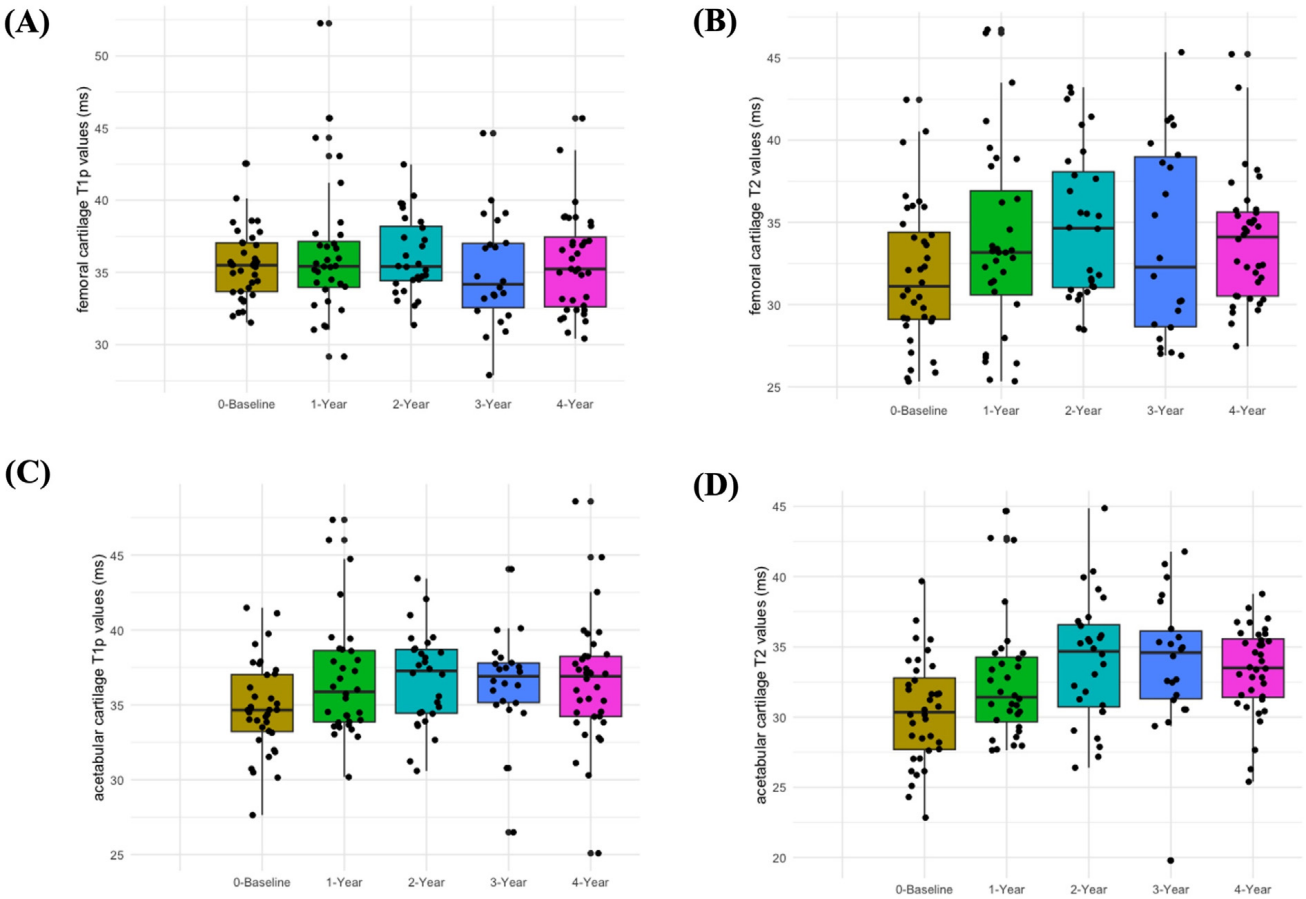
Box and Whisker plots illustrating the femur cartilage mean $T_{1\rho \;}$ and $T_{2}$ values(A) and (B) and acetabular cartilage mean $T_{1\rho \;}$ and $T_{2}$ values(C) and (D) at various time points(baseline, 1st-year, 2nd-year, 3rd-year and 4th-year follow-up).

In the longitudinal variability study, all the eighteen subjects data were processed and evaluated successfully. Based on the KL-score assessment, 17 hips were in the KL-score 0 group, 15 hips were in the KL-score 1 group, 3 hips were in the KL-score 2 group, and 1 hip was in the KL-score 3 group. Fig. 5 represents the Box-and-Whisker plots of all the hip-OA patients mean $T_{1\rho \;}$ and $T_{2}$ values of the femur and acetabular cartilage in the longitudinal study.

**Supplementary figure. 2:**
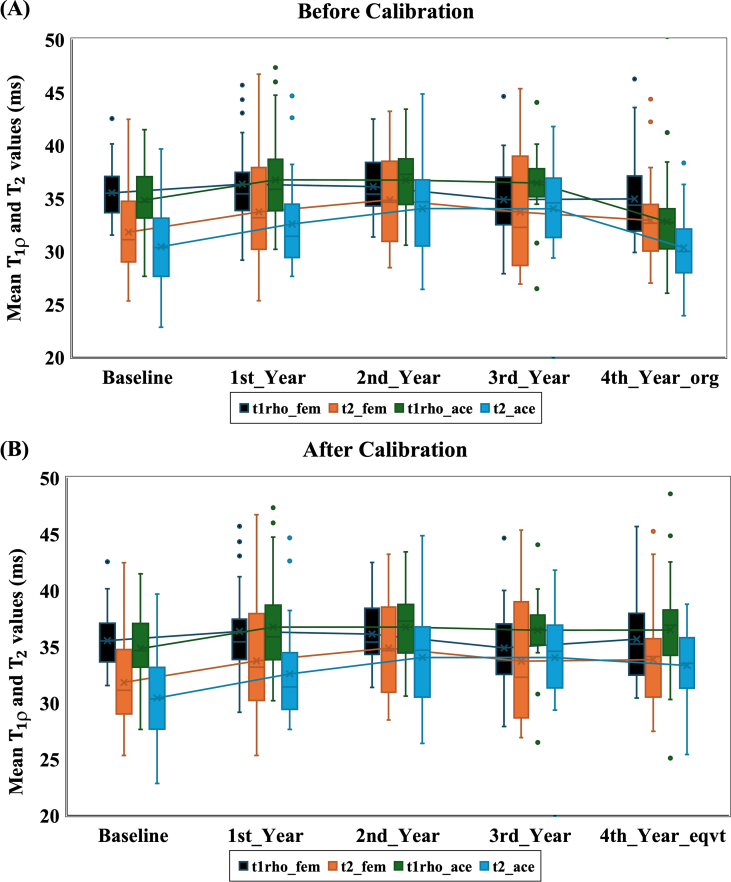
Box and Whisker plots show the upward changes in cartilage after calibration. Image(A) shows the mean $T_{1\rho \;}$ and values of the femur and acetabular cartilage region from baseline to 4th year follow-up scans(without calibration). Image(B) shows the mean $T_{1\rho \;}$ and $T_{2}$ values of the femur and acetabular cartilage after calibration.

The Box-and-Whisker plots in the Supplementary Fig. 2 demonstrated an upward trajectory of $T_{1\rho \;}$ and $T_{2}$ values after calibration (Supplementary Fig. 2(B)) during the 4th year follow-up. The PC analysis results showed that 52 ​% of subjects in the longitudinal variability study have a positive trend in the mean $T_{1\rho \;}$ values of the femur cartilage after calibration correction, whereas 42 ​% showed the same trend without calibration. Similarly, 48 ​% of subjects showed increased $T_{2}$ femur cartilage after calibration, whereas 44 ​% showed the same trend without calibration. The results showed that 52 ​% of subjects have an increasing trend in the mean $T_{1\rho \;}$ values of the acetabular cartilage after calibration, whereas only 11 ​% showed the same trend without calibration. 38 ​% of the subjects showed an increase in the $T_{2}$ acetabular cartilage after calibration, whereas 19 ​% showed the same trend without calibration. The percentage change analysis observed that the $T_{1\rho \;}$ and $T_{2}$ values increased after calibration more on the acetabular cartilage.

LME model analysis revealed significant correlations between variables and cartilage regions. As a predictor variable, BMI significantly correlated with the femur and acetabular cartilage, as shown in Table 2. Acquisition time points exhibited positive correlations with mean $T_{2}$ values of femoral cartilage and mean $T_{1\rho \;}$ and $T_{2}$ values of the acetabular cartilage. Female sex was significantly correlated with mean $T_{1\rho \;}$ values of femoral cartilage.

**Table 2:** LME results based on mean $T_{1\rho \;}$ and $T_{2}$ values of the whole femur and acetabular cartilage region. Variables and cartilage regions showing significant correlations are presented in this table. BMI: body mass index, Sex: Male or Female, Acq_year: Longitudinal study time points.

$T_{1\;}$**and**$T_{2}$**values**	**Value**	**Std.Error**	**DF**	**t-value**	**p-value**	**Parameter**
$T_{1\;}$_Femur	1.19	0.14	115	8.79	<0.0001^∗^	BMI
	4.76	1.60	35	2.98	0.0052^∗^	Sex
$T_{2}$_Femur	1.18	0.16	115	7.53	<0.0001^∗^	BMI
	2.56	0.90	115	2.85	0.0052^∗^	Acq_year
$T_{1\;}$_Acet	1.41	0.11	115	12.37	<0.0001^∗^	BMI
	2.03	0.88	115	2.30	0.0230^∗^	Acq_year
$T_{2}$_Acet	1.27	0.12	115	10.45	<0.0001^∗^	BMI
	4.04	0.97	115	4.18	0.0001^∗^	Acq_year

∗p-value <0.05.

In subregion analysis, BMI was significantly correlated with mean $T_{1\rho \;}$ and $T_{2}$ values across various subregions, including $R_{2}$-$R_{6}$ of the femur cartilage and $R_{2}$-$R_{5}$ of the acetabular cartilage (Table 3). Female sex was significantly correlated with femoral mean $T_{1\rho \;}$ values in $R_{2}$, $R_{3}\;\text{and}\;R_{4}$ regions, while it correlated with femoral mean $T_{2}$ values in the $R_{2}$ region only. Similarly, the acetabular mean $T_{1\rho \;}$ and $T_{2}$ mean values in the $R_{2}$ subregion showed significant correlations with female sex. $T_{1\rho \;}$ mean values of femoral subregion $R_{3}$ were significantly correlated with the acquisition year time point, whereas mean $T_{2}$ values of femoral subregions $R_{2}$ and $R_{3}\;\text{were}$ correlated with the acquisition year. In the acetabular region, mean $T_{1\rho \;}$ values of $R_{5}$ were significantly correlated, while mean $T_{2}$ values of subregions $R_{2}-$ $R_{5}$ showed significant correlations with the acquisition year. Additionally, the KL-score demonstrated a significant correlation with mean $T_{1\rho \;}$ and $T_{2}$ values of the femoral $R_{2}$ subregion.

**Table 3:** LME results based on $T_{1\rho \;}$ and $T_{2}$ cartilage mean values of femur ($R_{2}$-$R_{6}$) and acetabular ($R_{2}$-$R_{5}$) cartilage subregions. Variables and cartilage regions showing significant correlations are presented in this table. BMI: body mass index, Sex: Male or Female, Acq_year: Longitudinal study time points, KL-score: *Kellgren-Lawrence score*.

	Subregions	Value (beta)	Std.Error	DF	t-value	p-value	Parameter
**Mean**$T_{1\rho \;}$**Values**	Femur $R_{2}$	1.18	0.17	115	6.86	<0.0001	BMI
		7.17	1.86	35	3.86	0.0005	Sex
		3.46	1.22	115	2.84	0.0053	KL -score
	Femur $R_{3}$	1.45	0.15	115	9.42	<0.0001	BMI
		4.03	1.69	35	2.38	0.021	Sex
		2.92	1.07	115	2.74	0.0071	Acq_year
	Femur $R_{4}$	1.22	0.16	115	7.85	<0.0001	BMI
		4.25	1.76	35	2.41	0.0211	Sex
	Femur $R_{5}$	1.55	0.16	115	9.42	<0.0001	BMI
	Femur $R_{6}$	1.27	0.14	115	9.13	<0.0001	BMI
Mean $T_{2\;}$ Values	Femur $R_{2}$	2.85	1.21	115	2.35	0.0204	KL-score
		1.08	0.17	115	6.41	<0.0001	BMI
		6.31	1.81	35	3.49	0.0013	Sex
		5.54	1.36	115	4.06	0.0001	Acq_year
	Femur $R_{3}$	0.74	0.20	115	3.80	0.0002	BMI
		3.19	1.08	115	2.96	0.0038	Acq_year
	Femur $R_{4}$	1.29	0.17	115	7.67	<0.0001	BMI
	Femur $R_{5}$	1.33	0.18	115	7.23	<0.0001	BMI
	Femur $R_{6}$	1.09	0.20	115	5.57	<0.0001	BMI

	**LME results of acetabular (**$R_{2}$**-**$R_{5}$**) cartilage subregions**						**Parameter**
	**Subregions**	**Value (beta)**	**Std.Error**	**DF**	**t-value**	**p-value**	

**Mean**$T_{1\rho \;}$**values**	Acetabular $R_{2}$	0.97	0.133	115	7.24	<0.0001	BMI
		4.5**7**	1.43	35	3.19	0.0030	Sex
	Acetabular $R_{3}$	1.69	0.13	115	13.40	<0.0001	BMI
	Acetabular $R_{4}$	1.43	0.15	115	9.53	<0.0001	BMI
	Acetabular $R_{5}$	1.46	0.15	115	9.84	<0.0001	BMI
		7.16	1.42	115	9.84	<0.0001	Acq_year
**Mean**$T_{2\;}$**values**	Acetabular $R_{2}$	0.83	0.14	115	5.92	<0.0001	BMI
		3.33	1.49	35	2.24	0.0317	Sex
		5.65	1.27	115	4.44	<0.0001	Acq_year
	Acetabular $R_{3}$	1.38	0.13	115	10.37	<0.0001	BMI
		4.61	1.18	115	3.90	0.0002	Acq_year
	Acetabular $R_{4}$	1.27	0.15	115	8.32	<0.0001	BMI
		3.05	1.20	115	2.55	0.0121	Acq_year
	Acetabular $R_{5}$	1.37	0.15	115	9.40	<0.0001	BMI
		4.87	1.25	115	3.89	0.0002	Acq_year

## 4 Discussion

In our 5-year follow-up study, the associations between cartilage $T_{1\rho \;}$ and $T_{2}$ relaxation times and parameters such as BMI, sex, KL-score and acquisition time points were established. Our longitudinal variability study used a second scanner (Scanner-2) to acquire the 4th-year follow-up images. The ROI and VBR analyses showed a statistically significant difference in mean cartilage $T_{1\rho \;}\text{and}$ $T_{2}$ values between the scanners based on an extra calibration study. A Z-score normalization method incorporated with the VBR technique was implemented to address the inter-scanner variability inherent in a multi-center study with unilateral and bilateral protocols. With our patient study, the LME analysis showed a notable correlation between BMI and the $T_{1\rho \;}$ and $T_{2}$ values of hip cartilage in both the femur and acetabular region. Furthermore, we observed a significant increase in acetabular $T_{1\rho \;}$ and $T_{2}$ values, as well as femoral $T_{2}$ values over the time points. As a result, our research builds upon previous investigations in hip cartilage compositional analysis, reinforcing $T_{1\rho \;}$ and $T_{2}$ values as valuable biomarkers for hip cartilage degradation, providing support for its use in multi-center studies through careful calibration and analytical approach [11].

The present longitudinal study conducted image acquisition unilaterally using Scanner-1 and 4th-year follow-up bilaterally using Scanner-2. Our group shows the preliminary results of comparing bilateral hip acquisition using Scanner-2 with unilateral acquisition using Scanner-1 [23]. The findings indicated that the simultaneous bilateral MAPSS acquisition technique reduced total imaging time by 28.41 ​% compared to two sequential unilateral hip acquisitions. Another reported study showed that the recently developed VBR analysis could be a more accurate tool for the local pattern evaluation of cartilage biochemical changes [14]. VBR aligns all subjects in a group to a single reference space, which serves as the basis for this technique. The findings from the VBR analysis align with the conventional ROI analysis. Also, studies showed that for reducing the inter-scanner variability, the scaling factor correction was done on quantified parameters using the Z-score based normalization technique [17,19]. In the calibration study, over 20 ​% of voxels exhibited a significant difference in $T_{1\rho \;}$ values and more than 10 ​% of voxels showed significant differences in $T_{2}$ values. However, after applying the Z-score based scaling factor, the VBR results showed that only less than 3.3 ​% of the voxels showed a significant difference. Hence, in the current study, this normalization technique was effectively utilized to mitigate inter-scanner variability. Based on these results, we recommend that adopting the Z-score based normalization method in future longitudinal studies can have the ability to reduce the inter-scanner variability, potentially enhancing the interpretability and applicability of $T_{1\rho \;}$ and $T_{2}$ mapping results in clinical practice and multicenter longitudinal research studies.

Based on our patient study, BMI was the prime characteristic correlated with the $T_{1\rho }$ and $T_{2}$ values in the femur, acetabular cartilage regions and its subregions. The onset and progression of hip osteoarthritis can be attributed to irregular mechanical stress resulting from increased weight on weight-bearing joints [24]. Generally, the biochemical changes in hip cartilage were quantitatively assessed through measurements of $T_{1\rho }$ and $T_{2}$ values. A prior study indicated that BMI was significantly increased in acetabular cartilage lesion progressors(p ​= ​0.05) and showed a higher mean $T_{1\rho }$ and $T_{2}$ values in the femoral cartilage of the lesion group at 18 months [11]. Another study reported that $T_{2}$ values of cartilage in meniscal tear patient data were significantly increased in an obese group compared to a normal BMI group(p ​= ​0.008) [25]. So, the positive correlation between biochemical changes of cartilage and BMI from our study showed the use of quantitative MR imaging for finding the progression of hip-OA [11,25]

In addition to BMI, sex, acquisition year time point and KL-score also showed a positive correlation with some femur and acetabular cartilage subregions. This correlation was consistent with the previous longitudinal study result, which showed that age, BMI and female sex had a positive trend of progression [11]. Subburaj et al. reported regional differences in individuals with and without femoroacetabular impingement, particularly observing significant differences in the anterior-superior region of the hip joint cartilage between patients and healthy subjects [26]. Another study showed that the anterior-superior region of the acetabulum was associated with increased hip contact stress in a finite element analysis [27]. Wyatt et al. recently highlighted that elevated $T_{1\rho }$ and $T_{2}$ values were detected in the anterior-superior and central regions of the acetabular cartilage in individuals with radiographic hip-OA [13]. Gallo et al. reported higher $T_{1\rho }$ and $T_{2}$ in the superoposterior region of the femoral cartilage for subjects with cartilage lesions over an 18-month follow-up [11]. Consistent with these findings, our study revealed that the $T_{1\rho \;}$ values of posterior-superior and $T_{2}$ values of posterior, posterior-superior subregions of femoral cartilage showed a statistically significant correlation with acquisition year. Also, the $T_{1\rho \;}$ values of anterior-superior subregion and $T_{2}$ values of posterior, posterior-superior, superior and anterior-superior subregions of acetabular cartilage showed a statistically significant correlation with acquisition year. The $T_{1\rho \;}$ and $T_{2}$ values are derived from different relaxation mechanisms; however, both parameters effectively examine the slow motion of water protons [28,29]. Therefore, the increasing trend of both $T_{1\rho \;}$ and $T_{2}$ values in cartilage over time may indicate hip cartilage degradation, showing their potential as valuable biomarkers for hip cartilage health.

There are limitations to this study that need to be taken into consideration, including a relatively small study population. Subsequent studies involving larger cohorts and extended follow-up periods can provide further substantiation for the results obtained in this study. Second, the atlas-based segmentation and VBR analysis were only applied to the four center slices selected from each hip. The cartilage segmentation and post-processing tools need to be developed based on the newly developed deep learning methods to mitigate this limitation. Third, the 4 ​mm slice thickness of MAPSS sequence might cause the partial volume effect during the quantitative analysis. Finally, the current study performed a calibration study in a single vendor(GE) multi-site scanner. Additional studies are required to determine if these same techniques could be applied to scale data between vendor scanners.

In summary, the calibration approach showcased the effectiveness of VBR and Z-score normalization in mitigating inter-scanner variability. The LME analysis revealed a significant correlation between BMI and $T_{1\rho }$ and $T_{2}$ values in the femur and acetabular cartilage over the 5-year follow-up study. Additionally, $T_{1\rho }$ and $T_{2}$ values of the posterior-superior region of the femur and anterior-superior region of acetabular cartilage exhibited significant increases over time. These results affirm the significance of increasing trends in $T_{1\rho }$ and $T_{2}$ relaxation times as biomarkers for assessing treatment efficacy in patients with hip-OA.

## Author contributions

**Rafeek Thahakoya:** Conceptualization, Investigation, Data Acquisition, Methodology, Software, Validation, Formal analysis, Data curation, Writing - Original draft, review & editing.

**Koren E. Roach, Rupsa Bhattacharjee:** Conceptualization, Investigation, Methodology, Software, Writing - Review & editing.

**Misung Han:** Investigation, Data Acquisition, Methodology**,** Validation, Writing - Review & editing.

**Fei Jiang:** Software, Formal analysis, Writing - Review & editing.

**Johanna Luitjens:** Investigation, Validation, Writing - Review & editing.

**Emma Bahroos:** Investigation, Validation, Writing - Review & editing.

**Valentina Pedoia, Richard B. Souza, Sharmila Majumdar:** Conceptualization, Investigation, Methodology, Supervision, Funding acquisition, Writing - Review & editing.

All authors approved the final version of the article.

## Role of the funding source

Research reported in this publication was supported by the 10.13039/100000069National Institute of Arthritis and Musculoskeletal and Skin Diseases of the 10.13039/100000002National Institutes of Health under award numbers: R01AR069006, K24AR072133.

## Declaration of competing interest

The authors have declared that no conflict of interest exists.
